# Subjects Which Will Be Taught and Demonstrated to Every Smith, on Receiving Instruction at the New Veterinary Forge

**Published:** 1818

**Authors:** James Carver


### SUBJECTS

### WHICH WILL BE TAUGHT AND DEMONSTRATED

### TO EVERY SMITH,

### ON RECEIVING INSTRUCTION

### AT THE NEW VETERINARY FORGE.

1. An Introductory Lecture, giving a general view of the nature of the Shoeing Art.

2. The views commonly entertained of the Shoeing Art, and causes of its defects by various characters, *supposed* to have a knowledge of these things.

3. Reasonings founded on the natural foot, but are irrelevant on the foot being shod.

4. Various principles of shoeing, as they are called, examined. Good and bad shoeing pointed out.

5. One principle only—to follow Nature as near as we can; that defined, and how to be obtained.

6 Fitting shoes, and driving nails, discretional circumstances only pointed out.

7. The difference of the foot after shoeing observed, with a series of nine years experiments, for accurately ascertaining the effects of the shoe after being shod; by B. Clarke, F.L.S. and V.S.

8. A variety of different experiments on the foot of the living horse, explained, during my residence at the College.

9. A description of the foot and hoof of the horse, in which their true nature is endeavoured to be established, not merely as a defence for the foot, but as a non-resisting machinery for the exertions of the animal, and repose of the weight, pointed out and explained.

10. Of the heels; apparent offices of the heels, as elastic beds for the weight of the animal, explained and pointed out.

11. The extraordinary state of the foal's foot, which does not obtain its full development until the fifth year, explained and pointed out.

12. How the weight is received and distributed over the basis of the foot, exhibited, explained, and pointed out.

13. Wall of the foot described; its curious termination in the centre of the foot; explained and pointed out by dissection. The bars, as elastic processes, also defined.

14. Of the frog; how a space in the foot is provided for it by nature, as the elastic key-stone of the foot, demonstrated by dissection. Also the cleft of the frog, and the frog-stay described.

15. Cushion of the frog and its uses, described and demonstrated by dissection.

16. An extraordinary hitherto undescribed part, the conoary frog band, pointed out by dissection.

17. The frog-stay, described by dissection; the rupture of that organ described and pointed out as the real cause of running thrush, by B. Clark, V. S.

18. The frog shown to possess the power of maintaining its own figure, and the curious doctrine of shoeing-smiths, in this respect, pointed out.

19. Their reasons for cutting the frog explained, and the remarkable interruption to the growth of it, from that cause, pointed out.

20. How ascertained; its causes suggested; and the cutting of it unnecessary, nearly in all cases. The frequent cause of ragged frogs pointed out for the above cause. The natural full grown frog never ragged, if never cut, pointed out.

21. The singular effect of shoeing on the frog described; with its natural exfoliations, considered and explained.

22. The different degrees of pressure the frog ought to receive when in health, and when in a state of disease, pointed out.

23. The sole; its singular mechanism exhibited by dissection.

24. Thickens by shoeing, and the wall also retarded and disturbed in its growth by shoeing, explained.

25. The horny and sensible lamina described, by dissection. Five hundred of the former surrounding the anterior surface of the wall, with five hundred of the latter plates of horn surrounding the posterior surface of the coffin bone, and coming in contact with each other, is shown to support the whole weight of the animal, proved by experiments at the Veterinary College.

26. The bearings of the natural hoof on the ground—its natural exfoliations, &c. pointed out—its natural form at five years old; as broad from heel to heel as from heel to toe, also explained—never of an oblong form in a state of nature, if never shod.

27. On standing in the stable—how it proves the entire destruction of the foot, for want of proper stable treatment and management.

28. On shoeing—on neat shoeing—on levelling the toe—on expanding of feet—how to be obtained; together with a closer examination of the nature of these things, pointed out and explained;—how and why the shoeing art has for so many ages been involved in a cloud of darkness. With conclusions how this branch of the Veterinary Art may be drawn from contempt to respectability.

*Note.*—The great evils produced by cutting, its causes and its remedies, together with a number of experiments on different horses, tried at the Veterinary College, explained and pointed out.

Dr. Carver deeming it of great importance to rectify and determine the weight of the shoe, recommends the following to the notice of the public.

It is a matter of astonishment to see some horses with shoes weighing each 3, 4, and 5 lbs., making together a burden of 12, 16, and 20 lbs. of iron attached to their four feet. It must be obvious to common sense, that such an additional weight, fixed to the extremities of the leg, must be productive of some inconvenience or other, by compelling the muscles and ligaments to greater exertion than necessary; beside other evil consequences, as the weight forcing out the nails, and thereby spoiling the texture of the crust, &c. Why then, we may ask, do not the shoeing smiths, who are daily impressed of these evils, and who are themselves the very authors of them, apply themselves to the correction of their own errors? The answer, I fear, is obvious; because *he who is uneducated*, and *destitute of sound principles* in his art, cannot turn to real profit the experience he has acquired, nor abandon the force of prejudice and custom in which he has so long journeyed; but satisfies himself to imitate and repeat only whatever he has seen or heard by others.

The weight of the shoes proposed by Dr. C. are as follow:

| | |
|---|---|
| 1. For wagon, cart, or dray horses, from | 2 lbs. to 2 lbs. 12 oz. |
| 2. For small horses of this kind, from | 1 lb. to 2 lbs. |
| 3. For largest size coach horses, from | 1 lb. to 1 lb. 12 oz. |
| 4. The small size do. do. from | 14 oz. to 1 lb. 4 oz. |
| 5. For large saddle horses, from | 1 lb. to 1 lb. 4 oz. |
| 6. For small size do. from | 12 oz. to 1 lb. |
| 7. For race horses, from | 3 to 5 oz. |

Dr. Carver's reasons for reducing the weight of shoes, as above stated, are as follow:

If a horse can be made to travel as safe, and as long, with a light shoe, of only a few ounces, why should pounds be added? By reducing the superfluous breadth of shoes, their thickness may be increased without making any addition to their weight.

I am, however, of the following opinion: that from the race horse to the cart horse, the same system of shoeing should be observed. The *size*, *thickness*, and *weight* of them *only*, should differ. The shoe of a race horse must, of course, be lighter than that of a saddle horse; a saddle horse lighter than that of a coach or bat horse; and these last, more so than that of a cart, wagon, or artillery horse. Shoes have, until lately, in almost every country, been made too heavy; but if the iron be good, and *well hammer-hardened*, until cold—a pound of iron worked in this way may be converted into half a pound of steel, and still answer all the purposes intended.

By adopting these few rules here laid down, you will, at least, avoid the grosser errors which are every day committed; and if you have sufficient courage to study a little more the necessity of acquiring the two principles of shoeing, by asking for advice and information from me, instead of being bigotted to the opinions of your stable friends, Tom, Dick, and Harry, who cannot possibly know any more about it than yourselves, public opinion will soon place you on a level with men who are enlightened, and therefore useful to society. I will observe upon the whole, that the less substance you take away from the natural defence of the foot, *but on particular occasions which may require it*, the less artificial defence will be necessary; therefore, the nearer you follow these few simple rules, the nearer will you all approach to perfection in an *art, which has, for so many ages, been involved in darkness.*

